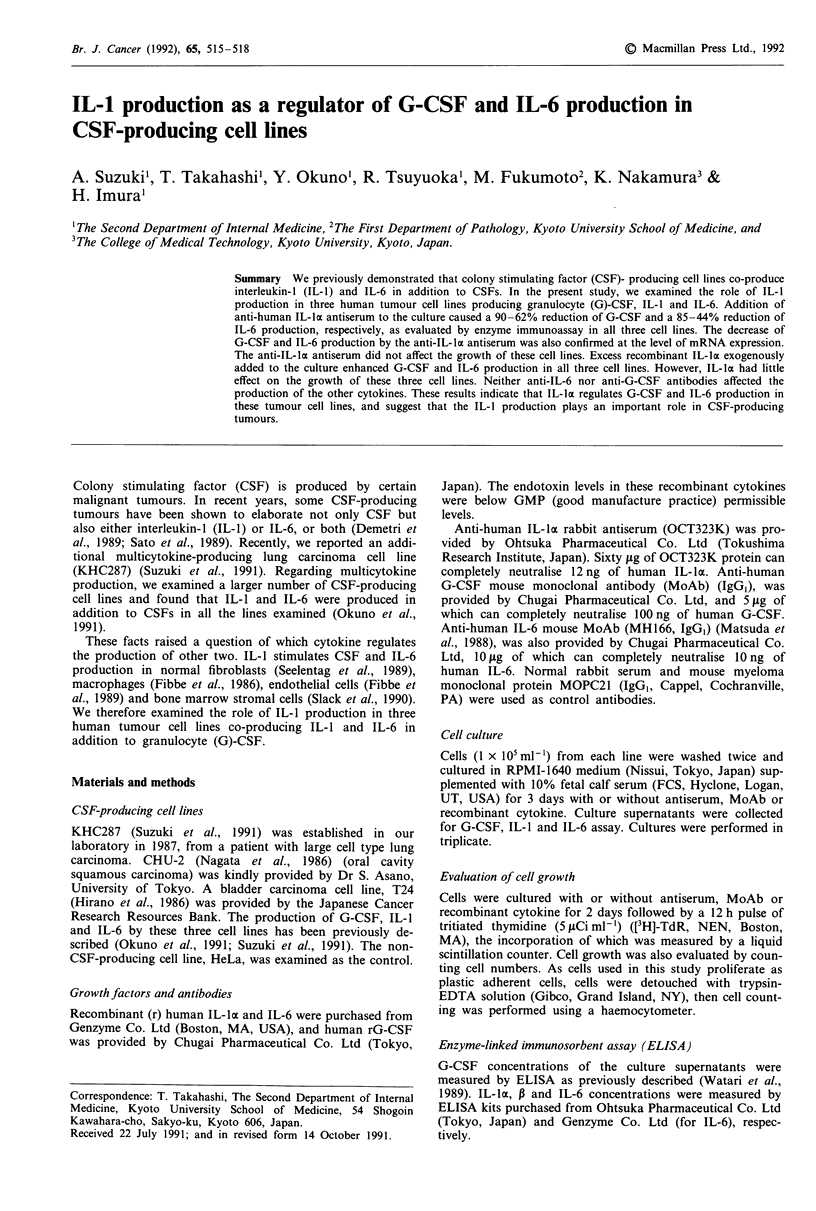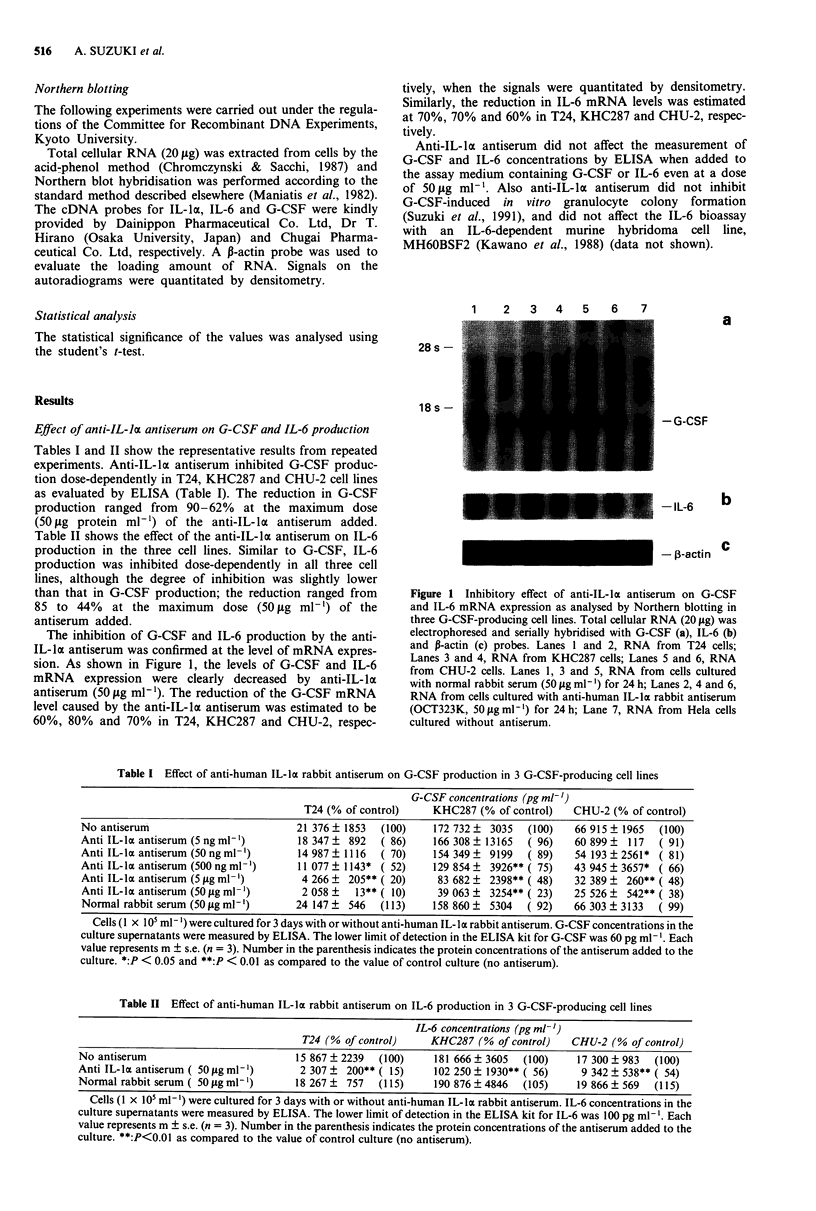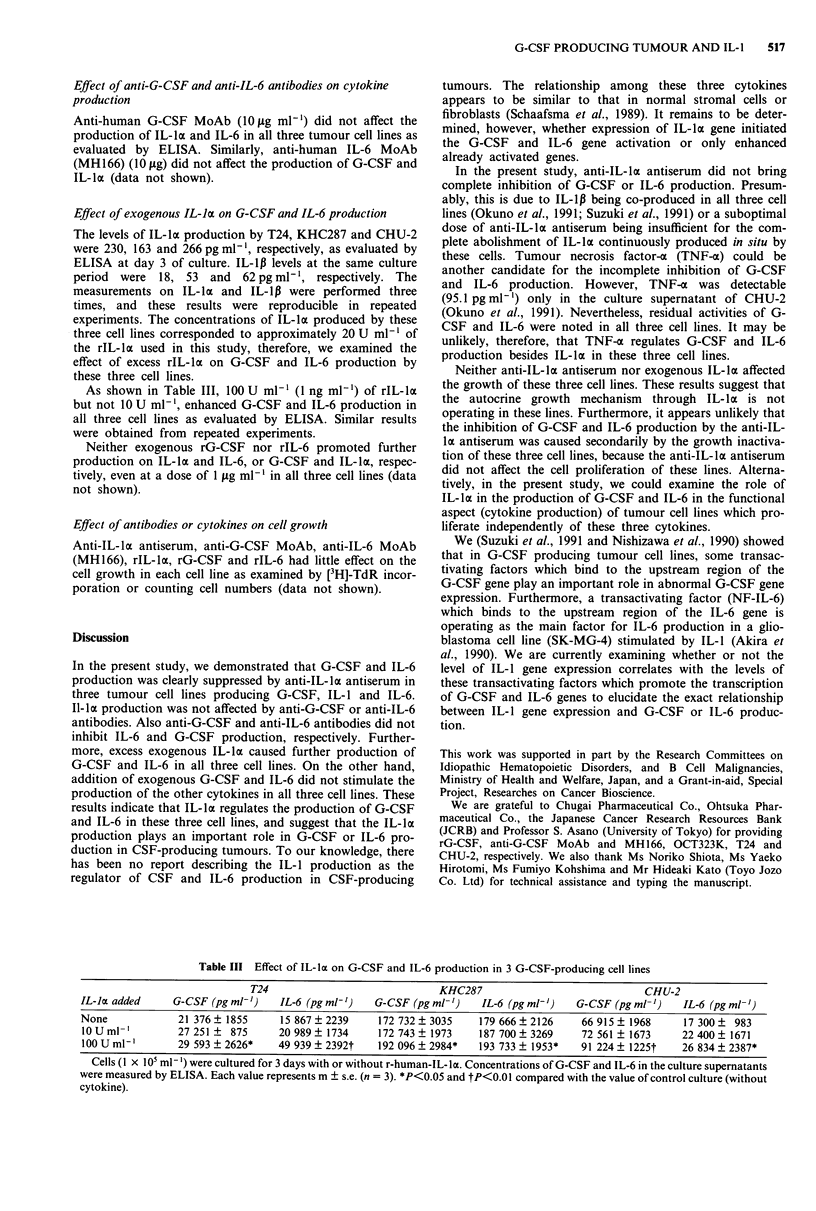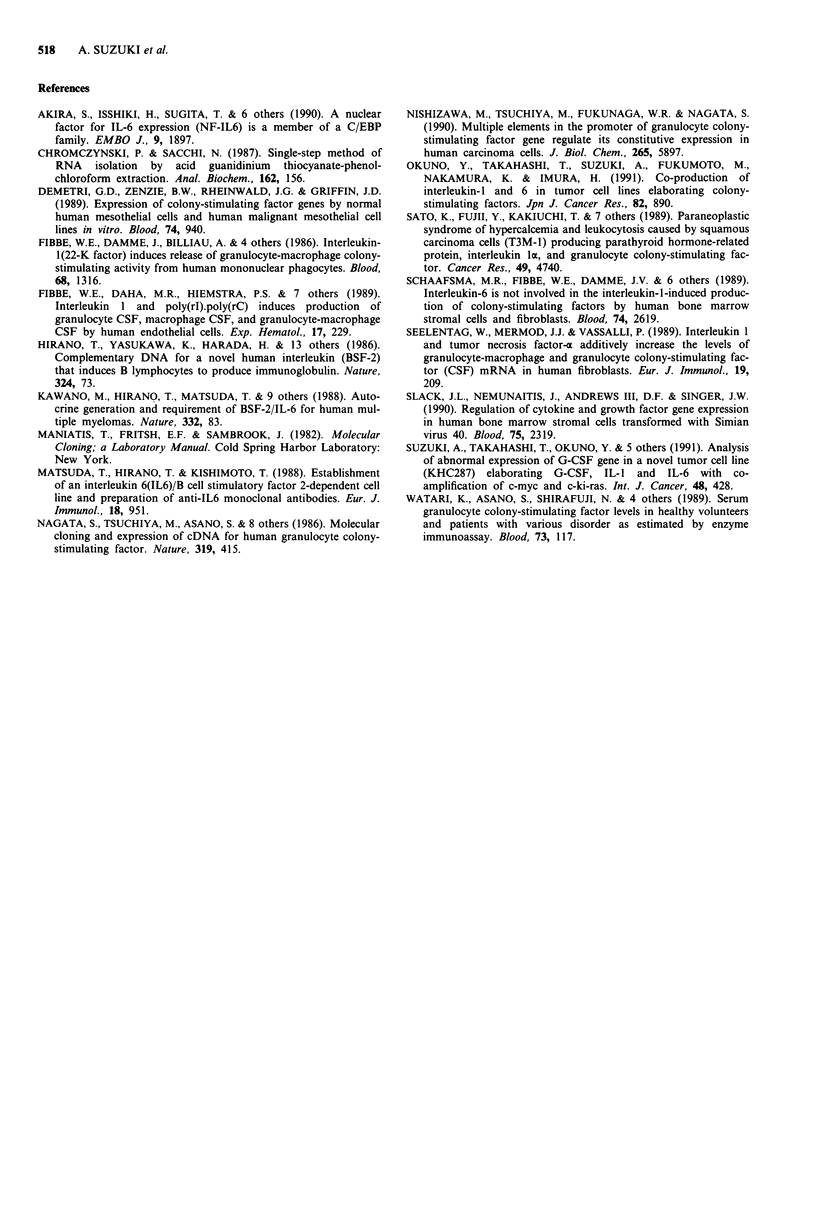# IL-1 production as a regulator of G-CSF and IL-6 production in CSF-producing cell lines.

**DOI:** 10.1038/bjc.1992.106

**Published:** 1992-04

**Authors:** A. Suzuki, T. Takahashi, Y. Okuno, R. Tsuyuoka, M. Fukumoto, K. Nakamura, H. Imura

**Affiliations:** Second Department of Internal Medicine, Kyoto University School of Medicine, Japan.

## Abstract

**Images:**


Br. J. Cancer (1992), 65, 515–518 © Macmillan Press Ltd., 1992

### IL-1 production as a regulator of G-CSF and IL-6 production in CSF-producing cell lines

A. Suzuki[1], T. Takahashi[1], Y. Okuno[1], R. Tsuyuoka[1], M. Fukumoto[2], K. Nakamura[3] & H. Imura[1]

[1]*The Second Department of Internal Medicine*, [2]*The First Department of Pathology, Kyoto University School of Medicine, and* [3]*The College of Medical Technology, Kyoto University, Kyoto, Japan.*

**Summary** We previously demonstrated that colony stimulating factor (CSF)- producing cell lines co-produce interleukin-1 (IL-1) and IL-6 in addition to CSFs. In the present study, we examined the role of IL-1 production in three human tumour cell lines producing granulocyte (G)-CSF, IL-1 and IL-6. Addition of anti-human IL-1α antiserum to the culture caused a 90–62% reduction of G-CSF and a 85–44% reduction of IL-6 production, respectively, as evaluated by enzyme immunoassay in all three cell lines. The decrease of G-CSF and IL-6 production by the anti-IL-1α antiserum was also confirmed at the level of mRNA expression. The anti-IL-1α antiserum did not affect the growth of these cell lines. Excess recombinant IL-1α exogenously added to the culture enhanced G-CSF and IL-6 production in all three cell lines. However, IL-1α had little effect on the growth of these three cell lines. Neither anti-IL-6 nor anti-G-CSF antibodies affected the production of the other cytokines. These results indicate that IL-1α regulates G-CSF and IL-6 production in these tumour cell lines, and suggest that the IL-1 production plays an important role in CSF-producing tumours.

Colony stimulating factor (CSF) is produced by certain malignant tumours. In recent years, some CSF-producing tumours have been shown to elaborate not only CSF but also either interleukin-1 (IL-1) or IL-6, or both (Demetri *et al.*, 1989; Sato *et al.*, 1989). Recently, we reported an additional multicytokine-producing lung carcinoma cell line (KHC287) (Suzuki *et al.*, 1991). Regarding multicytokine production, we examined a larger number of CSF-producing cell lines and found that IL-1 and IL-6 were produced in addition to CSFs in all the lines examined (Okuno *et al.*, 1991).

These facts raised a question of which cytokine regulates the production of other two. IL-1 stimulates CSF and IL-6 production in normal fibroblasts (Seelentag *et al.*, 1989), macrophages (Fibbe *et al.*, 1986), endothelial cells (Fibbe *et al.*, 1989) and bone marrow stromal cells (Slack *et al.*, 1990). We therefore examined the role of IL-1 production in three human tumour cell lines co-producing IL-1 and IL-6 in addition to granulocyte (G)-CSF.

### Materials and methods

### *CSF-producing cell lines*

KHC287 (Suzuki *et al.*, 1991) was established in our laboratory in 1987, from a patient with large cell type lung carcinoma. CHU-2 (Nagata *et al.*, 1986) (oral cavity squamous carcinoma) was kindly provided by Dr S. Asano, University of Tokyo. A bladder carcinoma cell line, T24 (Hirano *et al.*, 1986) was provided by the Japanese Cancer Research Resources Bank. The production of G-CSF, IL-1 and IL-6 by these three cell lines has been previously described (Okuno *et al.*, 1991; Suzuki *et al.*, 1991). The non-CSF-producing cell line, HeLa, was examined as the control.

### *Growth factors and antibodies*

Recombinant (r) human IL-1α and IL-6 were purchased from Genzyme Co. Ltd (Boston, MA, USA), and human rG-CSF was provided by Chugai Pharmaceutical Co. Ltd (Tokyo, Japan). The endotoxin levels in these recombinant cytokines were below GMP (good manufacture practice) permissible levels.

Anti-human IL-1α rabbit antiserum (OCT323K) was provided by Ohtsuka Pharmaceutical Co. Ltd (Tokushima Research Institute, Japan). Sixty μg of OCT323K protein can completely neutralise 12 ng of human IL-1α. Anti-human G-CSF mouse monoclonal antibody (MoAb) ($IgG_1$), was provided by Chugai Pharmaceutical Co. Ltd, and 5 μg of which can completely neutralise 100 ng of human G-CSF. Anti-human IL-6 mouse MoAb (MH166, $IgG_1$) (Matsuda *et al.*, 1988), was also provided by Chugai Pharmaceutical Co. Ltd, 10 μg of which can completely neutralise 10 ng of human IL-6. Normal rabbit serum and mouse myeloma monoclonal protein MOPC21 ($IgG_1$, Cappel, Cochranville, PA) were used as control antibodies.

### *Cell culture*

Cells ($1 \times 10^5$ $ml^{-1}$) from each line were washed twice and cultured in RPMI-1640 medium (Nissui, Tokyo, Japan) supplemented with 10% fetal calf serum (FCS, Hyclone, Logan, UT, USA) for 3 days with or without antiserum, MoAb or recombinant cytokine. Culture supernatants were collected for G-CSF, IL-1 and IL-6 assay. Cultures were performed in triplicate.

### *Evaluation of cell growth*

Cells were cultured with or without antiserum, MoAb or recombinant cytokine for 2 days followed by a 12 h pulse of tritiated thymidine (5 μCi $ml^{-1}$) ([$^3$H]-TdR, NEN, Boston, MA), the incorporation of which was measured by a liquid scintillation counter. Cell growth was also evaluated by counting cell numbers. As cells used in this study proliferate as plastic adherent cells, cells were detouched with trypsin-EDTA solution (Gibco, Grand Island, NY), then cell counting was performed using a haemocytometer.

### *Enzyme-linked immunosorbent assay (ELISA)*

G-CSF concentrations of the culture supernatants were measured by ELISA as previously described (Watari *et al.*, 1989). IL-1α, β and IL-6 concentrations were measured by ELISA kits purchased from Ohtsuka Pharmaceutical Co. Ltd (Tokyo, Japan) and Genzyme Co. Ltd (for IL-6), respectively.

Correspondence: T. Takahashi, The Second Department of Internal Medicine, Kyoto University School of Medicine, 54 Shogoin Kawahara-cho, Sakyo-ku, Kyoto 606, Japan.
Received 22 July 1991; and in revised form 14 October 1991.

*Northern blotting*

The following experiments were carried out under the regulations of the Committee for Recombinant DNA Experiments, Kyoto University.

Total cellular RNA (20 μg) was extracted from cells by the acid-phenol method (Chromczynski & Sacchi, 1987) and Northern blot hybridisation was performed according to the standard method described elsewhere (Maniatis *et al.*, 1982). The cDNA probes for IL-1α, IL-6 and G-CSF were kindly provided by Dainippon Pharmaceutical Co. Ltd, Dr T. Hirano (Osaka University, Japan) and Chugai Pharmaceutical Co. Ltd, respectively. A β-actin probe was used to evaluate the loading amount of RNA. Signals on the autoradiograms were quantitated by densitometry.

*Statistical analysis*

The statistical significance of the values was analysed using the student's *t*-test.

### Results

*Effect of anti-IL-1α antiserum on G-CSF and IL-6 production*

Tables I and II show the representative results from repeated experiments. Anti-IL-1α antiserum inhibited G-CSF production dose-dependently in T24, KHC287 and CHU-2 cell lines as evaluated by ELISA (Table I). The reduction in G-CSF production ranged from 90–62% at the maximum dose (50 μg protein ml$^{-1}$) of the anti-IL-1α antiserum added. Table II shows the effect of the anti-IL-1α antiserum on IL-6 production in the three cell lines. Similar to G-CSF, IL-6 production was inhibited dose-dependently in all three cell lines, although the degree of inhibition was slightly lower than that in G-CSF production; the reduction ranged from 85 to 44% at the maximum dose (50 μg ml$^{-1}$) of the antiserum added.

The inhibition of G-CSF and IL-6 production by the anti-IL-1α antiserum was confirmed at the level of mRNA expression. As shown in Figure 1, the levels of G-CSF and IL-6 mRNA expression were clearly decreased by anti-IL-1α antiserum (50 μg ml$^{-1}$). The reduction of the G-CSF mRNA level caused by the anti-IL-1α antiserum was estimated to be 60%, 80% and 70% in T24, KHC287 and CHU-2, respectively, when the signals were quantitated by densitometry. Similarly, the reduction in IL-6 mRNA levels was estimated at 70%, 70% and 60% in T24, KHC287 and CHU-2, respectively.

Anti-IL-1α antiserum did not affect the measurement of G-CSF and IL-6 concentrations by ELISA when added to the assay medium containing G-CSF or IL-6 even at a dose of 50 μg ml$^{-1}$. Also anti-IL-1α antiserum did not inhibit G-CSF-induced *in vitro* granulocyte colony formation (Suzuki *et al.*, 1991), and did not affect the IL-6 bioassay with an IL-6-dependent murine hybridoma cell line, MH60BSF2 (Kawano *et al.*, 1988) (data not shown).

**Figure 1** Inhibitory effect of anti-IL-1α antiserum on G-CSF and IL-6 mRNA expression as analysed by Northern blotting in three G-CSF-producing cell lines. Total cellular RNA (20 μg) was electrophoresed and serially hybridised with G-CSF (**a**), IL-6 (**b**) and β-actin (**c**) probes. Lanes 1 and 2, RNA from T24 cells; Lanes 3 and 4, RNA from KHC287 cells; Lanes 5 and 6, RNA from CHU-2 cells. Lanes 1, 3 and 5, RNA from cells cultured with normal rabbit serum (50 μg ml$^{-1}$) for 24 h; Lanes 2, 4 and 6, RNA from cells cultured with anti-human IL-1α rabbit antiserum (OCT323K, 50 μg ml$^{-1}$) for 24 h; Lane 7, RNA from Hela cells cultured without antiserum.

**Table I** Effect of anti-human IL-1α rabbit antiserum on G-CSF production in 3 G-CSF-producing cell lines

| | *G-CSF concentrations (pg ml$^{-1}$)* | | |
|---|---|---|---|
| | T24 (% of control) | KHC287 (% of control) | CHU-2 (% of control) |
| No antiserum | 21 376 ± 1853 (100) | 172 732 ± 3035 (100) | 66 915 ± 1965 (100) |
| Anti IL-1α antiserum (5 ng ml$^{-1}$) | 18 347 ± 892 ( 86) | 166 308 ± 13165 ( 96) | 60 899 ± 117 ( 91) |
| Anti IL-1α antiserum (50 ng ml$^{-1}$) | 14 987 ± 1116 ( 70) | 154 349 ± 9199 ( 89) | 54 193 ± 2561* ( 81) |
| Anti IL-1α antiserum (500 ng ml$^{-1}$) | 11 077 ± 1143* ( 52) | 129 854 ± 3926** ( 75) | 43 945 ± 3657* ( 66) |
| Anti IL-1α antiserum (5 μg ml$^{-1}$) | 4 266 ± 205** ( 20) | 83 682 ± 2398** ( 48) | 32 389 ± 260** ( 48) |
| Anti IL-1α antiserum (50 μg ml$^{-1}$) | 2 058 ± 13** ( 10) | 39 063 ± 3254** ( 23) | 25 526 ± 542** ( 38) |
| Normal rabbit serum (50 μg ml$^{-1}$) | 24 147 ± 546 (113) | 158 860 ± 5304 ( 92) | 66 303 ± 3133 ( 99) |

Cells (1 × 10$^5$ ml$^{-1}$) were cultured for 3 days with or without anti-human IL-1α rabbit antiserum. G-CSF concentrations in the culture supernatants were measured by ELISA. The lower limit of detection in the ELISA kit for G-CSF was 60 pg ml$^{-1}$. Each value represents m ± s.e. ($n = 3$). Number in the parenthesis indicates the protein concentrations of the antiserum added to the culture. *:$P < 0.05$ and **:$P < 0.01$ as compared to the value of control culture (no antiserum).

**Table II** Effect of anti-human IL-1α rabbit antiserum on IL-6 production in 3 G-CSF-producing cell lines

| | *IL-6 concentrations (pg ml$^{-1}$)* | | |
|---|---|---|---|
| | *T24 (% of control)* | *KHC287 (% of control)* | *CHU-2 (% of control)* |
| No antiserum | 15 867 ± 2239 (100) | 181 666 ± 3605 (100) | 17 300 ± 983 (100) |
| Anti IL-1α antiserum ( 50 μg ml$^{-1}$) | 2 307 ± 200** ( 15) | 102 250 ± 1930** ( 56) | 9 342 ± 538** ( 54) |
| Normal rabbit serum ( 50 μg ml$^{-1}$) | 18 267 ± 757 (115) | 190 876 ± 4846 (105) | 19 866 ± 569 (115) |

Cells (1 × 10$^5$ ml$^{-1}$) were cultured for 3 days with or without anti-human IL-1α rabbit antiserum. IL-6 concentrations in the culture supernatants were measured by ELISA. The lower limit of detection in the ELISA kit for IL-6 was 100 pg ml$^{-1}$. Each value represents m ± s.e. ($n = 3$). Number in the parenthesis indicates the protein concentrations of the antiserum added to the culture. **:$P$<0.01 as compared to the value of control culture (no antiserum).

*Effect of anti-G-CSF and anti-IL-6 antibodies on cytokine production*

Anti-human G-CSF MoAb (10 µg $ml^{-1}$) did not affect the production of IL-1α and IL-6 in all three tumour cell lines as evaluated by ELISA. Similarly, anti-human IL-6 MoAb (MH166) (10 µg) did not affect the production of G-CSF and IL-1α (data not shown).

*Effect of exogenous IL-1α on G-CSF and IL-6 production*

The levels of IL-1α production by T24, KHC287 and CHU-2 were 230, 163 and 266 pg $ml^{-1}$, respectively, as evaluated by ELISA at day 3 of culture. IL-1β levels at the same culture period were 18, 53 and 62 pg $ml^{-1}$, respectively. The measurements on IL-1α and IL-1β were performed three times, and these results were reproducible in repeated experiments. The concentrations of IL-1α produced by these three cell lines corresponded to approximately 20 U $ml^{-1}$ of the rIL-1α used in this study, therefore, we examined the effect of excess rIL-1α on G-CSF and IL-6 production by these three cell lines.

As shown in Table III, 100 U $ml^{-1}$ (1 ng $ml^{-1}$) of rIL-1α but not 10 U $ml^{-1}$, enhanced G-CSF and IL-6 production in all three cell lines as evaluated by ELISA. Similar results were obtained from repeated experiments.

Neither exogenous rG-CSF nor rIL-6 promoted further production on IL-1α and IL-6, or G-CSF and IL-1α, respectively, even at a dose of 1 µg $ml^{-1}$ in all three cell lines (data not shown).

*Effect of antibodies or cytokines on cell growth*

Anti-IL-1α antiserum, anti-G-CSF MoAb, anti-IL-6 MoAb (MH166), rIL-1α, rG-CSF and rIL-6 had little effect on the cell growth in each cell line as examined by [$^3$H]-TdR incorporation or counting cell numbers (data not shown).

### Discussion

In the present study, we demonstrated that G-CSF and IL-6 production was clearly suppressed by anti-IL-1α antiserum in three tumour cell lines producing G-CSF, IL-1 and IL-6. Il-1α production was not affected by anti-G-CSF or anti-IL-6 antibodies. Also anti-G-CSF and anti-IL-6 antibodies did not inhibit IL-6 and G-CSF production, respectively. Furthermore, excess exogenous IL-1α caused further production of G-CSF and IL-6 in all three cell lines. On the other hand, addition of exogenous G-CSF and IL-6 did not stimulate the production of the other cytokines in all three cell lines. These results indicate that IL-1α regulates the production of G-CSF and IL-6 in these three cell lines, and suggest that the IL-1α production plays an important role in G-CSF or IL-6 production in CSF-producing tumours. To our knowledge, there has been no report describing the IL-1 production as the regulator of CSF and IL-6 production in CSF-producing tumours. The relationship among these three cytokines appears to be similar to that in normal stromal cells or fibroblasts (Schaafsma *et al.*, 1989). It remains to be determined, however, whether expression of IL-1α gene initiated the G-CSF and IL-6 gene activation or only enhanced already activated genes.

In the present study, anti-IL-1α antiserum did not bring complete inhibition of G-CSF or IL-6 production. Presumably, this is due to IL-1β being co-produced in all three cell lines (Okuno *et al.*, 1991; Suzuki *et al.*, 1991) or a suboptimal dose of anti-IL-1α antiserum being insufficient for the complete abolishment of IL-1α continuously produced *in situ* by these cells. Tumour necrosis factor-α (TNF-α) could be another candidate for the incomplete inhibition of G-CSF and IL-6 production. However, TNF-α was detectable (95.1 pg $ml^{-1}$) only in the culture supernatant of CHU-2 (Okuno *et al.*, 1991). Nevertheless, residual activities of G-CSF and IL-6 were noted in all three cell lines. It may be unlikely, therefore, that TNF-α regulates G-CSF and IL-6 production besides IL-1α in these three cell lines.

Neither anti-IL-1α antiserum nor exogenous IL-1α affected the growth of these three cell lines. These results suggest that the autocrine growth mechanism through IL-1α is not operating in these lines. Furthermore, it appears unlikely that the inhibition of G-CSF and IL-6 production by the anti-IL-1α antiserum was caused secondarily by the growth inactivation of these three cell lines, because the anti-IL-1α antiserum did not affect the cell proliferation of these lines. Alternatively, in the present study, we could examine the role of IL-1α in the production of G-CSF and IL-6 in the functional aspect (cytokine production) of tumour cell lines which proliferate independently of these three cytokines.

We (Suzuki *et al.*, 1991 and Nishizawa *et al.*, 1990) showed that in G-CSF producing tumour cell lines, some transactivating factors which bind to the upstream region of the G-CSF gene play an important role in abnormal G-CSF gene expression. Furthermore, a transactivating factor (NF-IL-6) which binds to the upstream region of the IL-6 gene is operating as the main factor for IL-6 production in a glioblastoma cell line (SK-MG-4) stimulated by IL-1 (Akira *et al.*, 1990). We are currently examining whether or not the level of IL-1 gene expression correlates with the levels of these transactivating factors which promote the transcription of G-CSF and IL-6 genes to elucidate the exact relationship between IL-1 gene expression and G-CSF or IL-6 production.

This work was supported in part by the Research Committees on Idiopathic Hematopoietic Disorders, and B Cell Malignancies, Ministry of Health and Welfare, Japan, and a Grant-in-aid, Special Project, Researches on Cancer Bioscience.

We are grateful to Chugai Pharmaceutical Co., Ohtsuka Pharmaceutical Co., the Japanese Cancer Research Resources Bank (JCRB) and Professor S. Asano (University of Tokyo) for providing rG-CSF, anti-G-CSF MoAb and MH166, OCT323K, T24 and CHU-2, respectively. We also thank Ms Noriko Shiota, Ms Yaeko Hirotomi, Ms Fumiyo Kohshima and Mr Hideaki Kato (Toyo Jozo Co. Ltd) for technical assistance and typing the manuscript.

**Table III** Effect of IL-1α on G-CSF and IL-6 production in 3 G-CSF-producing cell lines

| | *T24* | | *KHC287* | | *CHU-2* | |
|---|---|---|---|---|---|---|
| *IL-1α added* | *G-CSF (pg $ml^{-1}$)* | *IL-6 (pg $ml^{-1}$)* | *G-CSF (pg $ml^{-1}$)* | *IL-6 (pg $ml^{-1}$)* | *G-CSF (pg $ml^{-1}$)* | *IL-6 (pg $ml^{-1}$)* |
| None | 21 376 ± 1855 | 15 867 ± 2239 | 172 732 ± 3035 | 179 666 ± 2126 | 66 915 ± 1968 | 17 300 ± 983 |
| 10 U $ml^{-1}$ | 27 251 ± 875 | 20 989 ± 1734 | 172 743 ± 1973 | 187 700 ± 3269 | 72 561 ± 1673 | 22 400 ± 1671 |
| 100 U $ml^{-1}$ | 29 593 ± 2626* | 49 939 ± 2392† | 192 096 ± 2984* | 193 733 ± 1953* | 91 224 ± 1225† | 26 834 ± 2387* |

Cells (1 × $10^5$ $ml^{-1}$) were cultured for 3 days with or without r-human-IL-1α. Concentrations of G-CSF and IL-6 in the culture supernatants were measured by ELISA. Each value represents m ± s.e. ($n$ = 3). *$P$<0.05 and †$P$<0.01 compared with the value of control culture (without cytokine).

### References

AKIRA, S., ISSHIKI, H., SUGITA, T. & 6 others (1990). A nuclear factor for IL-6 expression (NF-IL6) is a member of a C/EBP family. *EMBO J.*, **9**, 1897.

CHROMCZYNSKI, P. & SACCHI, N. (1987). Single-step method of RNA isolation by acid guanidinium thiocyanate-phenol-chloroform extraction. *Anal. Biochem.*, **162**, 156.

DEMETRI, G.D., ZENZIE, B.W., RHEINWALD, J.G. & GRIFFIN, J.D. (1989). Expression of colony-stimulating factor genes by normal human mesothelial cells and human malignant mesothelial cell lines *in vitro*. *Blood*, **74**, 940.

FIBBE, W.E., DAMME, J., BILLIAU, A. & 4 others (1986). Interleukin-1(22-K factor) induces release of granulocyte-macrophage colony-stimulating activity from human mononuclear phagocytes. *Blood*, **68**, 1316.

FIBBE, W.E., DAHA, M.R., HIEMSTRA, P.S. & 7 others (1989). Interleukin 1 and poly(rI).poly(rC) induces production of granulocyte CSF, macrophage CSF, and granulocyte-macrophage CSF by human endothelial cells. *Exp. Hematol.*, **17**, 229.

HIRANO, T., YASUKAWA, K., HARADA, H. & 13 others (1986). Complementary DNA for a novel human interleukin (BSF-2) that induces B lymphocytes to produce immunoglobulin. *Nature*, **324**, 73.

KAWANO, M., HIRANO, T., MATSUDA, T. & 9 others (1988). Autocrine generation and requirement of BSF-2/IL-6 for human multiple myelomas. *Nature*, **332**, 83.

MANIATIS, T., FRITSH, E.F. & SAMBROOK, J. (1982). *Molecular Cloning; a Laboratory Manual*. Cold Spring Harbor Laboratory: New York.

MATSUDA, T., HIRANO, T. & KISHIMOTO, T. (1988). Establishment of an interleukin 6(IL6)/B cell stimulatory factor 2-dependent cell line and preparation of anti-IL6 monoclonal antibodies. *Eur. J. Immunol.*, **18**, 951.

NAGATA, S., TSUCHIYA, M., ASANO, S. & 8 others (1986). Molecular cloning and expression of cDNA for human granulocyte colony-stimulating factor. *Nature*, **319**, 415.

NISHIZAWA, M., TSUCHIYA, M., FUKUNAGA, W.R. & NAGATA, S. (1990). Multiple elements in the promoter of granulocyte colony-stimulating factor gene regulate its constitutive expression in human carcinoma cells. *J. Biol. Chem.*, **265**, 5897.

OKUNO, Y., TAKAHASHI, T., SUZUKI, A., FUKUMOTO, M., NAKAMURA, K. & IMURA, H. (1991). Co-production of interleukin-1 and 6 in tumor cell lines elaborating colony-stimulating factors. *Jpn J. Cancer Res.*, **82**, 890.

SATO, K., FUJII, Y., KAKIUCHI, T. & 7 others (1989). Paraneoplastic syndrome of hypercalcemia and leukocytosis caused by squamous carcinoma cells (T3M-1) producing parathyroid hormone-related protein, interleukin 1α, and granulocyte colony-stimulating factor. *Cancer Res.*, **49**, 4740.

SCHAAFSMA, M.R., FIBBE, W.E., DAMME, J.V. & 6 others (1989). Interleukin-6 is not involved in the interleukin-1-induced production of colony-stimulating factors by human bone marrow stromal cells and fibroblasts. *Blood*, **74**, 2619.

SEELENTAG, W., MERMOD, J.J. & VASSALLI, P. (1989). Interleukin 1 and tumor necrosis factor-α additively increase the levels of granulocyte-macrophage and granulocyte colony-stimulating factor (CSF) mRNA in human fibroblasts. *Eur. J. Immunol.*, **19**, 209.

SLACK, J.L., NEMUNAITIS, J., ANDREWS III, D.F. & SINGER, J.W. (1990). Regulation of cytokine and growth factor gene expression in human bone marrow stromal cells transformed with Simian virus 40. *Blood*, **75**, 2319.

SUZUKI, A., TAKAHASHI, T., OKUNO, Y. & 5 others (1991). Analysis of abnormal expression of G-CSF gene in a novel tumor cell line (KHC287) elaborating G-CSF, IL-1 and IL-6 with co-amplification of c-myc and c-ki-ras. *Int. J. Cancer*, **48**, 428.

WATARI, K., ASANO, S., SHIRAFUJI, N. & 4 others (1989). Serum granulocyte colony-stimulating factor levels in healthy volunteers and patients with various disorder as estimated by enzyme immunoassay. *Blood*, **73**, 117.